# Factors associated with low utilisation of cervical cancer screening among urban women in Lilongwe, Malawi: a cross sectional study

**DOI:** 10.1186/s12905-023-02324-0

**Published:** 2023-04-17

**Authors:** Patricia Kamanga, Bo Zhang, William Stones

**Affiliations:** 1 Department of Public Health, School of Public Health and Family Medicine, College of Medicine, University of Malawi, Blantyre Malawi, Private Bag 360, Blantyre, Malawi; 2 Food Safety and Health Research Center, School of Public Health, Southern Medical University, 511505 Shaitanan Road, Guangzhou, Guangdong China; 3 Kamuzu Central Hospital, P.O. Box 149, Lilongwe, Malawi

**Keywords:** Cervical cancer, Women, Screening, Visual inspection with acetic acid, Lilongwe City, Malawi

## Abstract

**Background:**

In 2012, more than half a million women (528,000) were diagnosed with cervical cancer around the world. More than 80% of cervical cancer occurs in developing nations, such as Malawi, where estimates of the disease’s burden show an incidence of 75.9 per 100,000 women and a mortality rate of 49.8 per 100,000 women (both age-adjusted). Despite its case fatality rate, cervical cancer can be avoided through immunization, early detection and screening. Malawi however, has low immunization and screening rates with coverage as low as 9% and 15%, respectively. Here our aim is to uncover factors that contribute to low utilization of cervical cancer screening services among women in Lilongwe, a large urban center.

**Methods:**

This was a qualitative cross-sectional study. Participants were chosen at random from a big metropolitan health center. In-depth interviews and two observations were undertaken by the researchers. Interviews were taped, transcribed verbatim, and content assessed.

**Results:**

A total of 24 women and 5 health workers, with an average age of 34.8 years, were questioned. 50% of women had completed secondary school, 33.3% had completed primary school, and 4% had completed no formal education. The majority of the women were housewives and entrepreneurs. 62.5% of the respondents had fewer than four children, 25% had four to six children, and 8.3% had more than six children. 91 − 6% of those surveyed were married, with 78% of Christians and 20% of Muslims. The majority of women were unaware of the importance of cervical cancer screening. Some people were concerned about marital troubles, pain during the process, “laziness,“ and the amount of time necessary. The majority of people would come for a test as a result of signs and symptoms. Male health personnel would be unable to screen Muslim women. All of the medical personnel had at least two years of experience. Women’s low involvement in cervical cancer screening has been linked by health workers to a lack of resources and a lack of community awareness.

**Conclusion:**

Cervical cancer can be prevented by early detection and treatment. Women, on the other hand, are uninformed about cervical cancer. Myths, misconceptions, cultural and religious beliefs, as well as service restrictions and community sensitization, influence the use of cervical cancer screening services. Addressing these issues has the potential to boost cervical cancer screening rates.

## Background

Most women in low-resource countries are motivated to seek medical treatment when they experience symptoms of cervical cancer, which frequently indicate that the illness has progressed to an advanced stage [1]. Interventions are suboptimal at this point, and outcomes are dismal. In low-resource settings, visual inspection with acetic acid (VIA) was implemented to detect precancerous alterations before invasive cervical cancer occurs [2], allowing for successful treatment. Despite VIA programming, many women with advanced cervical cancer still present to referral centers with limited treatment options [1].

Persistent infection with the human papillomavirus (HPV), particularly strains 16 and 18, causes cervical cancer [3]. Men can also be infected with the virus [3, 4]. It is one of the most common cancers in women, with estimates indicating that over a million women globally have the disease, with the majority of them lacking access to diagnostic and treatment services [3]. According to projections based on prediction modelling, 604,127 new cases of cancer will be identified worldwide in 2020, with 341,831 women dying from the disease [5]. Even in well-resourced health systems, social and demographic factors can be impediments to cervical cancer screening. In the United States, for example, the healthcare system and socioeconomic demographic variables impeded access to cervical cancer screening programs [6].

Cervical cancer is considered to be the most common malignancy among women in Sub-Saharan Africa, with 34.8% of new cases and 22.5% mortality per 100 000 women [7, 8]. In African countries, this amounts to an estimated 119,284 instances of cervical cancer each year, with 81,687 fatalities [9]. According to estimates, developing nations, especially Sub-Saharan Africa, account for 83% of all new cervical cancer cases each year and 85% of all cervical cancer deaths [8]. Unfortunately, in developing countries, there are numerous impediments to early detection and adequate treatment of cervical cancer [10]. 90% of cervical cancer deaths are thought to be owing to a lack of access to prevention and treatment [11], human and material resources, diagnostic tests or equipment, and treatment facilities are among the factors highlighted [10].

Cervical cancer is the most frequent cancer among Malawian women of reproductive age, with the highest incidence among women aged 40 years [12] and an age-standardized incidence of 72.9 per 100,000 in 2018 [12]. Cervical cancer is responsible for just under half of all cancer cases [12], with an 80% fatality rate [2]. Malawi’s population of at-risk women is predicted to be 4.50 million women aged 15 and up [2]. According to estimates, 3684 Malawian women are diagnosed with cervical cancer per year, with 2314 dying as a result [2]. According to a local survey, cervical cancer was the top cause of mortality among the five most common kinds of cancer, with a mean survival time of roughly 9 months [14].

Cervical cancer screening improves treatment outcomes by ensuring early diagnosis of malignancy [15–17]. In 1999, Malawi launched a cervical cancer prevention program [2]. Currently, VIA is used to screen for cervical cancer in 81 of 328 health clinics across 29 health districts [12]. Despite this, screening rates in the country are poor, and many women who are referred to the central hospital have the advanced disease [1]. This has been ascribed to a lack of awareness, a poor perception of susceptibility, and low perception of advantages, with signs of cervical cancer serving as triggers to action [2]. Anxiety, inadequate service delivery, and cultural and religious views were also mentioned as impediments [15–17].

In this case, we wanted to figure out what the current client and service health hurdles are to screening uptake in low-income urban areas.

## Methods

### Study design and population

This was a qualitative cross-sectional study conducted at Bwaila Hospital, a government hospital in Lilongwe, Malawi’s capital city, that serves both urban and rural people. The hospital, which is located in the heart of Lilongwe, offers both curative and preventive care, including cervical cancer screening. The facility was chosen for the study because it is the city’s sole government-owned hospital now offering cervical cancer screening services. Notably, Kamuzu Central Hospital, which serves as a local referral center, was left out because it does not offer cervical cancer screening. The district’s rural hospitals were not included in the current study, which focused on the urban setting. Similarly, while private urban institutions provide screening, the current research focused on low-income people whose primary access is through free government-mandated programs.

### Sample recruitment strategy

There were two sample categories, and these were: (i) women and (ii) health workers. According to WHO guidelines, women between the ages of 25 and 50 can be screened using visual inspection with ascetic acid (VIA) [18]. Therefore, in the first category we recruited women aged 25 to 50 years old living in Lilongwe’s urban regions. The women were sampled using purposive sampling methods. These women were recruited at the health facility in a private area to provide privacy. The second category included health workers who had worked for at least three months at Bwaila Hospital’s outpatient reproductive family health unit.

### Sample size

The health workers stated that they screen 17 to 20 women each day on average, which equates to 300 participants. In this study, however, saturation was attained at 24 participants. As a result, 24 women were interviewed. In the area of health professionals, there were two trained cervical cancer nurses and three additional health workers offering other reproductive health services, therefore, a total of five health workers were questioned.

### Data collection methods

In February 2017, the lead investigator collected data at Bwaila hospital’s family healthy unit. Data were obtained through in-depth interviews with women in the Chichewa language following a topic guide, self-administered questionnaires in English for health workers, and participant observation in the health facility utilizing methodological triangulation. Prior to finalization, the tools were tested at the same hospital, with changes made to improve item clarity and sequencing. The interviews were taped with a recorder and later transcribed and translated by the lead investigator. Following the recording and verification of the transcripts, audio recordings and any missing notes were incorporated. Data on service delivery was gathered through participant observation (equipment, attitude, space, health education). The focus of the observation was on the time it took to open the clinic and close it, as well as the procedure of screening the woman as she came in for the service.

### Data management and analysis

Data was verbatim transcribed and translated before being examined for content. The process of content analysis is systematically summarizing phrases or sentences while adhering to a classification scheme [19]. In this study, the lead investigator and an independent coder independently read the transcript several times to get the context. After familiarization with data, codes were developed, categorization and summary of verbal data was performed. After coding, the codes were classified into themes based on their commonalities. We used percentages to reach an agreement on the codes and reach on the final themes.

### Trustworthiness in qualitative research

The following evaluative criteria were used to establish trustworthiness in the study: credibility, dependability, transferability, and confirmability [20]. Persistent observation, triangulation, prolonged interaction with participants, pushing for further information; and the use of women who came for services at the family health unit and had a direct personal interest in the topic was used to establish credibility in this study. The interview guide was used to preserve consistency in the questions, resulting in dependability. The proper and detailed description of the study environment, as well as the inclusion and exclusion criteria, assured transferability. The sample size was also mentioned, as well as the data gathering techniques. Confirmability was verified by audio recording and note-taking during interviews.

### Data interpretation

**Fig. 1:**
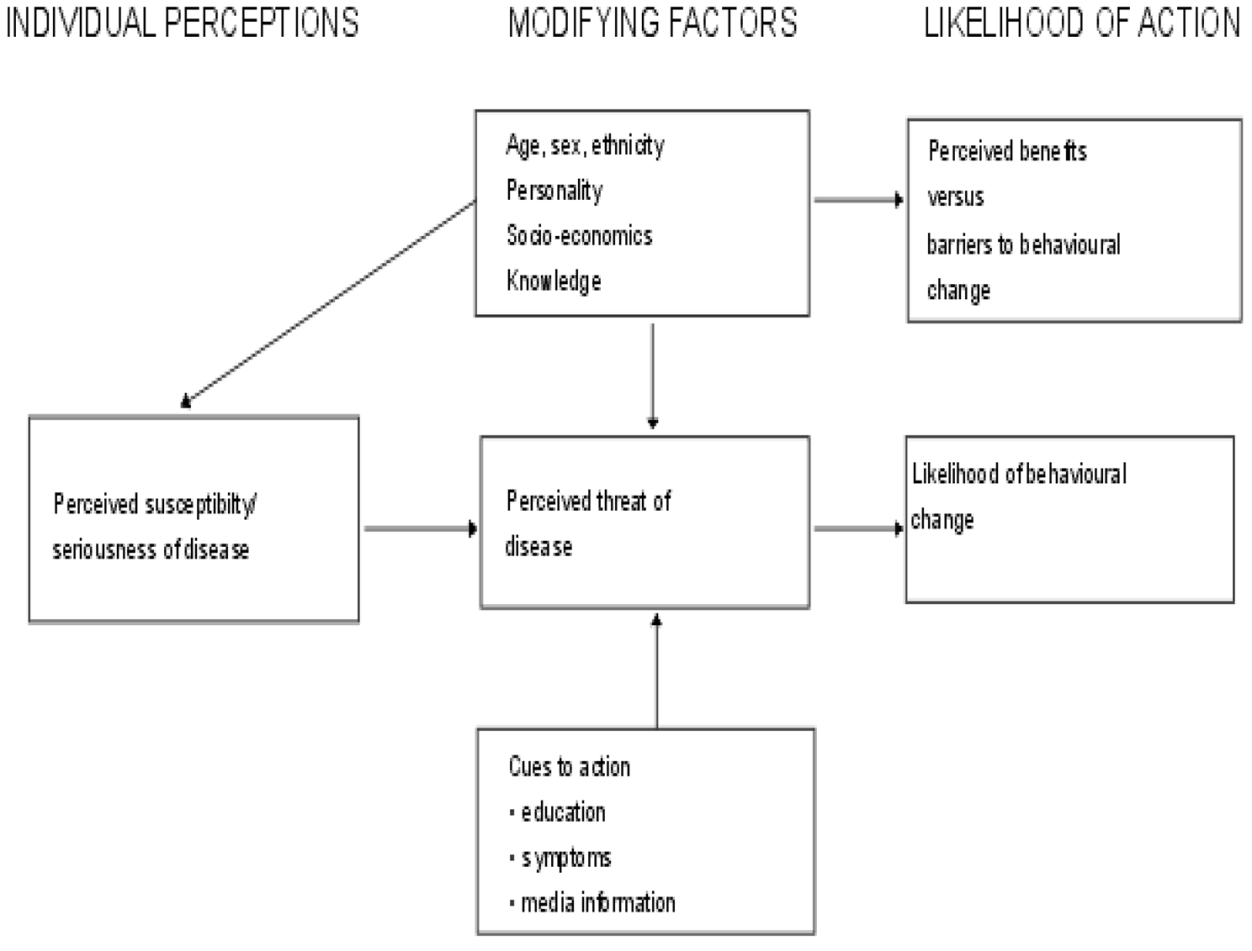
The Health Belief Model [21]

The research question was framed using the Health Belief Model (HBM) (see Fig. 1) [21]. This study focuses on individual attitudes and beliefs to explain and predict health behaviors [21]. The concept argues that an individual’s perception of susceptibility and severity of a health problem determines a threat, which increases the likelihood of taking preventative action in a health intervention that reduces the threat [21]. The model goes on to illustrate how modifying elements such as demographic, socio-psychological, and structural variables influence an individual’s perception of susceptibility, seriousness, advantages, and barriers to taking action on a health condition [21]. This is a preventive hypothesis, and it corresponds to the notion of cervical cancer screening as a preventive measure. Individual attitudes and beliefs are used to operationalize this [21]. Perceived advantages, perceived susceptibility, perceived restrictions, and perceived severity are the dimensions that the model focuses on [21]. People’s readiness to act is accounted for by these principles, which leads to the ability to exhibit self-efficacy [21]. According to the HBM, if a person believes that a negative health condition such as cervical cancer may be averted, he or she will take associated action (e.g., cervical cancer screening). Furthermore, the individual has a positive expectation that by taking a recommended health action, she will avoid a negative health condition, e.g., cervical cancer screening will be effective at preventing cervical cancer and believes that she can successfully take a recommended health action. That is if she can go for cervical cancer screening comfortably and confidently.

### Application of the model to the study

The HBM shows how the following factors can influence behavioral change in terms of health prevention:

(i) If a woman believes she is not at risk for cervical cancer, the chances of her taking preventive steps are minimal. It becomes difficult to voluntarily undergo cervical cancer screening.
(ii) The severity of cervical cancer (perceived severity); if a woman is unaware that cervical cancer is a dangerous disease that cannot be treated at any stage, she may refuse to have her cervical cancer screened. If she believes it is a serious condition, on the other hand, she will seek preventive services.
(iii) Women’s perceptions of impediments to cervical cancer screening services (perceived barriers); the challenges women have in obtaining cervical cancer screening services may influence their desire to undergo screening. Individual, cultural, religious, or service delivery variables may be involved.
(iv) Perceived benefits vs. behavioral change hurdle; It will be difficult for a woman to go for screening if she is unaware of the benefits of doing so. This could be due to a lack of understanding of the advantages of screening.

Age, sex, ethnicity, knowledge, status, and personality are all modifying factors. Some women may refuse to have their breasts screened because they believe they are too young or too old. Some people may feel uneasy about getting screened by a male health care practitioner. Some people may be unable to attend a socioeconomic screening because they lack transportation and understanding about cervical cancer.

(vi) Inducing factors (cues to action), such as education, symptoms, and media information. Some women may seek screening if they are experiencing symptoms of cervical cancer. Others may come because they were directed to do so by medical staff, and still others because they heard about it on the radio or at a health talk.

## Ethical considerations

To get informed consent, participants were provided with extensive information about the study’s objective, importance or benefits, potential hazards, and data collection processes. Participants were ensured of their privacy and anonymity; no names appeared on the topic guide paper, and respondents were not penalized for declining to participate in the study. The project received approval from the College of Medicine Research Ethics Committee (COMREC). P.11/16/2076 is the reference.

## Results

### Demographic characteristics of women

Table 1 below summarizes the demographic features of women. The research plan called for up to 30 women to participate, however, data collection reached saturation at 24. The women interviewed ranged in age from 25 to 49 years old, with an average age of 34.8 years.

**Table 1:** Demographic characteristics of participants (women) (n = 24)

Age	Frequency	Percentage
25–30 years	11	45.8
> 30–35 years	4	16.7
> 35–40 years	6	25.0
> 40–45 years	1	4.2
> 45–50 years	2	8.3
**Education level**		
None	3	12.5
Primary	8	33.3
Secondary	12	50.0
Tertiary	1	4.2
**Parity**		
0	1	4.2
1–3	15	62.5
4–6	6	25.0
7–9	2	8.3
**Marital status**		
Single	2	8.3
Married	22	91.6
**Religion**		
CCAP	10	41.7
Islam	5	20.8
Roman Catholic	4	16.7
Other	5	20.8
**Occupation**		
Business	9	37.5
Housewife	6	25.0
Employed	5	20.8
Farmer	4	16.7

### Qualitative results

The following themes and subthemes emerged:

#### Lack of education

i. Lack of knowledge
  The majority of women (75%) had little knowledge about cervical cancer or cervical cancer screening. The majority of people (79%) believed that cervical cancer may be cured at any stage and that doctors can work miracles. Some people (29%) believe it is a serious condition, while others (21%) do not believe it is. Some people (8%) have no idea what cervical cancer was.
ii. Misconceptions/myths
  The majority of women (42%) indicated they would accept malignant cells if they were discovered, and a few women (13%) said:
  *That means it is the end of my life and me because it cannot be cured* [R11, age 26].
  Several women (29%) believed that their marriage might break or have problems because of cervical cancer.
  *It disturbs marriages since women drain fluid and are in pain the husband will not agree to this and may end up going out, you know men are difficult* [R17, Age 37].
iii. Cues to action
  The majority (63%) indicated they would seek cervical cancer screening if they experienced signs and symptoms such as severe abdominal discomfort and abnormal vaginal discharge or bleeding. Some (21%) said they just came because their doctor informed them, they needed to come in for a screening to identify and know the diagnosis. The majority of women (46%) indicated they are unable to attend screening because they have heard it is an unpleasant procedure. Metal instruments are, after all, used.
  *I hear the screening uses metal instruments which when inserted in the vagina can cause trauma and pain* [ R05, age 28].
  Some (13%) said laziness while others (17%) lack time to come for screening.

#### Cultural/religious factors

Because of their faith and beliefs, all Muslim women (20.8%) claimed they could not let a male health care practitioner screen them. The women were unaware that at the time, all of the providers were women.

#### Lack of support from management

i. Shortage of resources
  Stocks of acetic acid were identified by all five health professionals as important issues, as were a lack of skilled health experts. In terms of physical assets;
  *We buy acetic acid on our own, despite presenting the issue to management.*
  In terms of human resources;
  *We are only two on daily basis to do the screening, which is a problem, but the bosses think we do nothing, they say there is no workload. “Furthermore, most health centers in Lilongwe urban stopped providing VIA due to lack of other resources like cryotherapy. As such this clinic is providing the service to the whole of Lilongwe urban”.*
ii. Community sensitization/awareness
  A lack of community sensitization on cervical cancer screening was mentioned by several of the nurses (40%). They used to go out into the community to raise awareness about cervical cancer, but these efforts have now been discontinued.

#### Hospital policies

The screening room was decent, and the personnel was pleasant, according to all of the women (100%). However, the majority of them (54%) reported that if they arrive after 9 a.m., they are sent back without being screened. It will be tough for them to return. Two participant observation sessions were held to observe and document people’s behavior, activities, and interactions in a methodical manner. The first observation focused on the working hours during weekdays. The clinic was open throughout the study period. According to observers, the clinic opened late at 9 o’clock in the morning, while women arrived earlier, and then closed at midday, yet the official working hours are from 8 o’clock in the morning to 4 o’clock in the afternoon. All women who arrived after the health education has been completed were asked to return the next day for screening. On average, 17 women were checked, with only five of them returning. The second observation was made during the screening process: the nurse collects all of the health passport booklets and provides a health presentation to the group of women about cervical cancer and screening. The health talk covered the concept of cervical cancer, its causes, and how to prevent it. All women who arrived after the books and education have been gathered and completed were sent back without being screened.

*I came at 7 a.m., I waited until 9 o’clock, then health education was given, and I was screened at 11 o’clock, imagine all these hours just for screening* [R08, age 40].

*I have been sent back because I came after the health education has already been done, so I have been told to come back next time and I should come before 9 [R14, age 44].*

## Discussion

Despite the country’s lengthy history of cervical screening, we discovered significant hurdles to uptake among highly educated women in this low-income urban environment. In comparison to older women, the data revealed that women between the ages of 25 and 35 (45.8%) came for screening more frequently. These findings contradict those of a study on cervical cancer screening clinic non-attendance, which revealed that young women are extremely busy and do not believe they are in danger of acquiring cervical cancer [22]. We also discovered that women with secondary school education outnumbered women who have completed secondary school and post-secondary education. In Zimbabwe, on the other hand, knowledge levels of cervical cancer were shown to be unrelated to educational levels [23]. The majority of the women were secondary school dropouts, yet there was no difference in knowledge between those with primary school and those with tertiary degrees.

Another Malawi study found that some women refuse to have their breasts tested because they don’t want to be screened by someone younger than them [24]. Younger women, on the other hand, avoided screening because they were afraid of discomfort and because the majority of them were still living with their parents and would be shocked if they went for a test [22]. While our sample was not randomly selected, almost all of the women in the current study had at least one child. This finding is consistent with a previous study that found that women with children were more likely to go for screening than women without children [22].

In terms of perceived severity, the majority of women thought cervical cancer could be cured at any stage. Furthermore, because women are unaware of the causes and predisposing factors, they believe it is not a significant problem. This may have contributed to women avoiding seeking help because they do not consider it to be a severe problem. As a result, they do not go for screening. This is consistent with the findings of research conducted in Mulanje, a rural district in Southern Malawi and South Africa, which revealed that lack of information and a low perceived threat were among the barriers to service access [2, 25]. Women in urban regions, on the other hand, were shown to be more knowledgeable than women in rural ones [26]. On the other hand, a few women believed that if they went for screening and were found to have cancerous cells, they would die or hurt their relationship with their husbands, which echoed another study in which women saw cervical cancer as a serious condition that could endanger their relationship with their partners and that the majority of people died [27].

Several studies have found psychological hurdles affecting women’s attendance, such as dread of the surgery, cancer diagnosis, and pain [22]. Misconceptions and myths such as dread of the operation, that it is unpleasant and traumatic, and fear of being diagnosed with cancer was shown to be barriers to accessing services in this study. This incorrect knowledge is spread by the ladies. This was also noted in a recent Malawi study, where existing misperceptions regarding cervical cancer screening programs, such as women ceasing to reproduce after having VIA and cryotherapy, hampered service delivery [24]. Another South African study found that some unscreened informants had heard from others that the treatment was painful and that it led women to walk about with their legs spread for a long [26]. Most women were also afraid that if their husbands found out they had cervical cancer, their marriage would end, and for those who were married, it would result in divorce.

According to prior research in Malawi [24], cultural and religious considerations created a challenge in women, particularly Muslim women, who prefer female providers to male providers to get the service. In other countries, such as Iran, a guideline mandates that only female providers, not male health workers, can screen a woman, ensuring that access to the service is not hampered by the presence of a male provider [28]. The presence of male physicians may affect women’s willingness to seek cervical cancer screening outside of the region and in other religious and cultural situations [29].

Service-side impediments to access include a lack of both human and material resources in clinics. A lack of provider monitoring was clear, with the clinic closing early and the screening not taking place until after 1 o’clock in the afternoon. These factors also contribute to low screening usage since if a woman comes for screening in the afternoon, she will be unable to access the program and will have to schedule another screening day, which is both costly and time-consuming for her. Inadequate human resources, lack of supervision, and frequent stock-outs of consumables and equipment have all been identified as challenges in Malawi’s health system [4].

The study found that community awareness initiatives have lapsed, leaving women unaware of the importance of cervical cancer screening. Low awareness of cervical cancer screening has also been noted as a hurdle in Ethiopia [30].

Opening times were one of the hurdles to screening in the current study: even in high-resource nations, clients may not be able to meet clinicians at convenient times [27]. This has prompted efforts to broaden access by allowing health workers to extend their working hours to accommodate all women who are unable to come during regular business hours [22].

### Implications for research and practice

Service gaps must be filled to ensure that services are accessible and affordable: even though they are free, taking time off from work has a significant opportunity cost, especially if the clinic is closed. To ensure wider access, those decentralized health clinics that are currently unable to provide services should be reactivated. To avoid gaps in supply, commodity supply, such as acetic acid supply, must be consistent. Finally, to ensure proper practice and conformity to service regulations, appropriate supervision of health professionals in cervical cancer clinics is required.

Women must be enabled to make their own decisions about cervical cancer screening and other preventive health services at the individual level. Women will be able to take control of their health in this way. Women should also be able to share preventive messages with their fellow women in their communities and encourage one another. According to the findings of the study, major campaigns on cervical cancer and screening are needed to educate women and the community because there is a significant dearth of information. With the help of community leaders, these efforts can be amplified.

### Future research

Research is needed to test different models of health communication that reflect population characteristics and realities and enable women to challenge and overcome service side barriers, such as advocating for convenient and accessible services.

### Study limitation

Due to the specific urban setting, the findings may not be nationally generalizable. Furthermore, the sample size was small, albeit in proportion to the scope of the study.

## Conclusion

Several factors were found to be associated with low utilization of cervical cancer screening services among urban women in Lilongwe, according to the study. Cultural and religious beliefs, a lack of knowledge about the severity of the disease and the availability of screening, time constraints for both women and health workers, myths and misconceptions among women, and a general lack of community sensitization are among these.

## Data Availability

The raw data are available from the corresponding author upon request for ethical approval.

## Abbreviations

CC: Cervical Cancer
HPV: Human Papilloma Virus
VIA: Visual Inspection with Acetic acid
WHO: World Health Organization
HIV: Human Immunodeficiency Virus
DHO: District Health Officer
HBM: Health Belief Model.

## References

[1] The Malawi government. Ministry of Health. National cervical cancer control strategy 2016–2020. Lilongwe

[2] Fort VK, sue Makin M, Aaron J, Ault K (2011). Barriers to cervical cancer screening in Mulanje, Malawi: a qualitative study. Patients Prefer and Adherence.

[3] World Health Organization (2006). Reproductive Health, World Health Organization. Chronic diseases, Health Promotion. Comprehensive cervical cancer control: a guide to essential practice.

[4] Maseko FC, Chirwa ML, Muula AS. Health systems challenges in cervical cancer prevention program in Malawi.Global health action. 2015 Dec1; 8(1):26282. 10.3402/gha.v8.26282. 10.3402/gha.v8.26282 PMC4306748 25623612

[5] World Health Organization (WHO)., International Agency on Research Cancer, 2020.Cervix –Uteri-Global Cancer Observatory.

[6] Wang X, Fang C, Tan Y, Liu A, Ma GX. Evidence-based intervention to reduce access barriers to cervical cancer screening among underserved Chinese American women. Journal of Women’s health 2010 Mar1: 19(3):463–9. 10.1089/jwh.1422. 10.1089/jwh.2009.1422 PMC2867551 20156089

[7] World Health Organization, International Agency for Research on Cancer. Prevention of cervical cancer through screening using visual inspection with acetic acid (VIA) and treatment with cryotherapy. A demonstration project in six African countries: Malawi, Madagascar, Nigeria, Uganda, the United Republic of Tanzania, and Zambia. Geneva:WHO; 2012.

[8] Ferlay J, Soerjomataram I, Dikshit R, Eser S, Mathers C, Rebelo M et al. Cancer incidence and mortality worldwide: sources, methods, and major patterns in GLOBOCAN 2012. International Journal of cancer. 2015 Mar1; 136(5):359–386. 10.1002/ijc.29210. 10.1002/ijc.29210 25220842

[9] Human Papilloma Virus and related diseases Report. 2019. www.hpv.net. Accessed 29 December 2020.

[10] Denny L, Anorlu R. Cervical cancer in Africa. Cancer epidemiology and prevention Biomarkers.2006 May 31; 21(9):1434–1438. 10.1158/1055-9965.EPI-12-0334. 10.1158/1055-9965.EPI-12-0334 22806169

[11] World Health Organisation (WHO)., / Africa/Health topics/cervical cancer. www.afro.who.int/health-topics/cervical-cancer. Accessed on 29 December 2020.

[12] Msyambozya KP, Dzamalala C, Mdokwe C, Kamiza S, Lemerani M, Dzowela T, Kathyola D. Burden of cancer in Malawi; common types, incidence, and trends: national population-based cancer registry. BMC research notes. 2012 Mar16; 5(1):1. 10.1186/1756-0500-5-149. 10.1186/1756-0500-5-149 PMC3327635 22424105

[13] World cancer research fund. Cervical cancer statistics. www.Wcrf/diet and cancer. Accessed on 29 December 2020.

[14] Msyambozya KP, Manda G, Tembo B, Thambo C, Chitete L, Mindiera C et al. Cancer survival in Malawi: a retrospective cohort study. The Pan African Medical Journal. 2014 Oct31;19. 10.11604/pamj.2014.19.234.4675 10.11604/pamj.2014.19.234.4675 PMC4377240 25838862

[15] Sankaranarayanan R, Rajikumar R, Theresa R, Esmy PO, Mahe C, Bagyalakshmi KR et al. Initial results from randomized trial of cervical visual screening in rural south India. International Journal of Cancer. 2004 April10; 109(3):461–7. 10.1002/ijc.11726 10.1002/ijc.11726 14961588

[16] Mbarara SU, Ikpeze OC, Okonkwo JE, Onyiaorah IV, Ukah CO. Knowledge, attitude, and practice of cervical cancer screening among women attending gynaecology clinics in a tertiary level medical care centre in South-eastern Nigeria. Journal of Reproductive Medicine. 2011 Nov1; 56(11):491.PMID: 22195332. 22195332

[17] Everett T, Bryant A, Griffin MF, Martin-Hirsch PP, Forbes CA, Jepson RG. Interventions targeted at women to encourage the uptake of cervical cancer screening. [Cochrane Database Syst Rev]. 2011 May 11 [cited 2017 July 13]. Available from: The Cochrane Library. 10.1002/14651858.CD002834.pub2 PMC4163962 21563135

[18] World Health. Organization (WHO) guidelines for screening and treatment of precancerous lesions for cervical cancer screening prevention. World Health Organization; 2013. 24716265

[19] Stemler S. An overview of content analysis, “practical assessment, research and evaluation. 2000. 10.7275/z6fm-2e34.

[20] Lincoln YS, Guba EG (1985). Naturalistic Inquiry.

[21] Green EC, Murphy E (2014). Health belief model. The Wiley Blackwell encyclopedia of health, illness, behaviour, and society.

[22] Waller J, Jackowska M, Marlow L, Wardle J. Exploring age differences in reasons for nonattendance for cervical screening: a qualitative study. BJOG: An International Journal of Obstetrics & Gynaecology. 2012 Jan 1; 119(1):26–32. 10.1111/j.1471-0528.2011.03030. x. 10.1111/j.1471-0528.2011.03030.x 21668764

[23] Mupepi SC, Sampselle CM, Johnson TR. Knowledge, attitudes, and demographic factors influencing cervical cancer screening behaviour of Zimbabwean women. Journal of Women’s Health. 2011 Jun1; 20(6):943–52. 10.1089/jwh.2010.2062. 10.1089/jwh.2010.2062 21671779

[24] Munthali AC, Ngwira BM, Taulo F. Exploring barriers to delivery of cervical cancer screening and early treatment services in Malawi: some views from service providers. Patient preference and adherence. 2015 Mar24; 9:501. 10.2147/PPA.S69286. 10.2147/PPA.S69286 PMC4376260 25848229

[25] Wellensiek N, Moodley M, Moodley J, Nkwanyana N. Knowledge of cervical cancer screening facilities among women from various socioeconomic backgrounds in Durban, Kwazulu Natal, South Africa. International Journal of Gynaecological Cancer. 2002; 12(4): 376 – 82. 10.1046/j.1525-1438.2002.01114. x. 10.1046/j.1525-1438.2002.01114.x 12144686

[26] Aswathy S, Queresh MA, Kurian B, Leelamoni K (2012). Cervical cancer screening: current knowledge and practice among women in rural population of Kenala, India. Indian J Med Res.

[27] Ma XG, Gao W, Fang CY, Tan Y, Feng Z, Ge S, An Nguyen J (2013). Health beliefs associated with cervical cancer screening among vietnamese Americans. J Women’s Health.

[28] Arkbari F, Shakibazadeh E, Pourreza A, Tavafian SS (2010). Barriers and facilitating factors for cervical cancer screening: a qualitative study from Iran. Iran J cancer Prev.

[29] Szarewski A, Cadman L, Ashdown-Barr L, Waller J (2009). Exploring acceptability of two self-sampling devices for human papillomavirus testing in cervical cancer screening context: a qualitative study of muslim women in London. J Med Sci.

[30] Gebru Z, Gerbaba M, Dirar A. Barriers to Cervical cancer screening in Arba Minch Town Ethiopia; a qualitative study. Journal of Community Medicine & Health Education. 2016 Feb29; 6:401. 10.4172/2161-0711.1000401.

